# Effects of Co-culture on Improved Productivity and Bioresource for Microalgal Biomass Using the Floc-Forming Bacteria *Melaminivora Jejuensis*

**DOI:** 10.3389/fbioe.2020.588210

**Published:** 2020-12-18

**Authors:** Dong-Hyun Kim, Hyun-Sik Yun, Young-Saeng Kim, Jong-Guk Kim

**Affiliations:** ^1^School of Applied Biosciences, Kyungpook National University, Daegu, South Korea; ^2^Department of Biology, College of Natural Sciences, Kyungpook National University, Daegu, South Korea; ^3^Research Institute of Ulleung-Do & Dok-Do, Kyungpook National University, Daegu, South Korea; ^4^School of Life Sciences and Biotechnology, BK21 Plus KNU Creative BioResearch Group, Kyungpook National University, Daegu, South Korea

**Keywords:** *Chlorella sorokiniana*, flocculation, *Melaminivora jejuensis*, sedimentation, biomass harvesting

## Abstract

Bacterial and algal floc formation was induced by inoculating three species of wastewater-derived bacteria (*Melaminivora jejuensis, Comamonas flocculans*, and *Escherichia coli*) into algal cultures (*Chlorella sorokiniana*). Bacterial and algal flocs formed in algal cultures inoculated with *M. jejuensis* and *C. flocculans*, and these flocs showed higher sedimentation rates than pure algal culture. The floc formed by *M. jejuensis* (4988.46 ± 2589.81 μm) was 10-fold larger than the floc formed by *C. flocculans* (488.60 ± 226.22 μm), with a three-fold higher sedimentation rate (*M. jejuensis*, 91.08 ± 2.32% and *C. flocculans*, 32.55 ± 6.33%). Biomass and lipid productivity were improved with *M. jejuensis* inoculation [biomass, 102.25 ± 0.35 mg/(L·day) and 57.80 ± 0.20 mg/(L·day)] compared with the productivity obtained under pure algal culture conditions [biomass, 78.00 ± 3.89 mg/(L·day) and lipids, 42.26 ± 2.11 mg/(L·day)]. Furthermore, the fatty acid composition of the biomass produced under pure algal culture conditions was mainly composed of C_16:0_ (43.67%) and C_18:2_ (45.99%), whereas the fatty acid composition of the biomass produced by *M. jejuensis* was mainly C_16:0_ (31.80%), C_16:1_ (24.45%), C_18:1_ (20.23%), and C_18:2_ (16.11%). These results suggest the possibility of developing an efficient method for harvesting microalgae using *M. jejuensis* and provide information on how to improve biomass productivity using floc-forming bacteria.

## Introduction

Microalgae are a source of bioenergy raw material that can be used to produce biofuels. This unique bioresource has been proposed as a solution to combat energy shortages and alleviate problems associated with global warming (Morales et al., 2019; Tan et al., 2019). Compared to terrestrial plants, microalgae have greater potential as a bioenergy raw material (Chen et al., 2020) with greater biomass productivity (Cooney et al., 2009; Chen et al., 2020). Typically, 10–20% of the biomass derived from microalgae consists of fatty acids that can be used as raw materials for bioenergy (Sajjadi et al., 2018). However, some limitations for industrial applications of microalgae bioenergy remain (Lee, 2001). The biomass produced through microalgae cultivation is harvested using processes such as centrifugation and filtration (Dassey and Theegala, 2013; Farooq et al., 2013). Significant losses and production costs are incurred during harvest (Dassey and Theegala, 2013; Farooq et al., 2013). Therefore, solutions to reduce the losses and production costs associated with harvesting processes are essential (Jonker and Faaij, 2013).

To reduce microalgae biomass production costs, methods have been developed to utilize wastewater to culture strains with high sedimentation rates (Cheng et al., 2019; Leite and Daniel, 2020). Multicellular strains with high sedimentation rates, such as those belonging to the *Pediastrum* genus, were proposed for this application (DA LUZ et al., 2002); however, culturing *Pediastrum* requires more time than that required for unicellular strains with lower sedimentation rates and biomass productivity, such as members of the *Chlorella* genus (Duygu et al., 2019; Stone et al., 2019). In addition, while the sedimentation rates of multicellular strains are relatively high, it takes a long time to harvest the sedimented biomass (Su et al., 2012). As a result of these sedimentation rate limitations, wastewater-based cultivation methods have also been investigated in an attempt to reduce the production costs associated with cultivation (Cheng et al., 2019). Wastewater contains inorganic materials required for microalgal growth such as nitrogen and phosphorous (Benítez et al., 2019). To purify wastewater, nitrogen, and phosphorous must be removed; thus, the cultivation of microalgae using wastewater can be used to simultaneously purify wastewater and produce biomass at a low cost (Benítez et al., 2019; Cheng et al., 2019). Furthermore, bacteria can be cultured together with microalgae during cultivation with wastewater (Kwon et al., 2019). Depending on the characteristics of the bacteria being cultured, bacteria-derived floc can often form. This bacteria-derived floc that forms contains microalgal cells, and the sedimentation properties of this floc are greater than those of the pure microalgal biomass. Therefore, the floc formed by bacteria can be applied to microalgal biomass harvesting (Wieczorek et al., 2015).

In this study, three bacterial strains [*Melaminivora jejuensis*, Korean Collection for Type Cultures (KCTC) 32230; *Comamonas flocculans*, KCTC 62943; *Escherichia coli*, DH5α] were inoculated into medium to form a floc with the microalgal strain *Chlorella sorokiniana* KNUA114. The floc-forming abilities of the inoculated strains were investigated and the sedimentation rates of the resulting biomass were compared. Gas chromatography/mass spectrometry (GC/MS) was used to determine the composition of fatty acid in biomass contained within each floc as a bioenergy raw material. Here, we demonstrated the application of floc-forming bacteria in microalgal harvesting and established the benefits for bioenergy development.

## Materials and Methods

### Whole Genome Analysis

The whole-genome analysis was applied to confirm the composition of bacterial floc-forming-related genes and the distribution of EPS genes that are expected to be related to flocculation with microalgae (Lee et al., 2013). *Melaminivora jejuensis* KCTC 32230 whole-genome sequencing data were assembled with PacBio SMRT Link 7.0.1 using the HGAP4 protocol (Pacific Biosciences, USA). PacBio RS II sequencing data were assembled with SMRT Portal 2.3.0 using the HGAP2 protocol (Pacific Biosciences, USA). The resulting contigs were circularized using Circlator 1.4.0 (Sanger Institute). Gene-finding and functional annotation pipeline of whole-genome assemblies were performed using the EzBioCloud genome database. Protein-coding sequences (CDSs) were predicted by Prodigal 2.6.2 (Hyatt et al., 2010). The CDSs were classified based on their roles, with reference to orthologous groups (EggNOG 4.5; http://eggnogdb.embl.de) (Powell et al., 2014). For more functional annotation, the predicted CDSs were compared with the Swissprot (Consortium, 2015), KEGG (Kanehisa et al., 2014), and SEED (Overbeek et al., 2005) databases using the UBLAST program (Edgar, 2010). The whole-genome shotgun BioProject accession number is PRJNA663428 with the locus tag prefix IDM45 (https://www.ncbi.nlm.nih.gov/bioproject/?term=txid1267217[Organism:~noexp]).

### Collection and Screening of Floc-Forming Bacterial Strains

Based on a previous study, we tried to select floc-forming bacteria, which are expected to form floc with microalgae, among strains derived from wastewater (Jimoh et al., 2019; Petrini et al., 2020). *C. flocculans* KCTC 62943 was isolated from a livestock wastewater treatment plant wastewater sample (52-109, Gyebaek-ro 499beon-gil, Chaeun-myeon, Nonsan-si, Chungcheongnam-do, 36°10′ 36.6″ N, 127°03′ 10.4″ E; Kim et al., 2019, 2020). *Melaminivora jejuensis* KCTC 32230, which was originally isolated from swine waste on Jeju Island, Republic of Korea (Kim et al., 2018), was obtained from the KCTC at the Korea Research Institute of Bioscience and Biotechnology, Korea. The isolation process was performed by streaking using R2A medium agar plates (with 15 g/L agarose; Reasoner and Geldreich, 1985). The isolated strains were identified using 16S rRNA gene sequencing (Wang and Qian, 2009). The floc-forming ability of the identified strains was screened; the flocs formed were observed and measured using an optical microscope (model BX53F; Olympus).

### Strains and Culture Conditions

*Melaminivora jejuensis* KCTC 32230 (Kim et al., 2018) and *C. flocculans* KCTC 62943 (Kim et al., 2019, 2020), which were clearly observed to form floc, were selected as the experimental strains. *E. coli*, which is related to floc and biofilm, was selected as the control strain. Three flocculation bacterial strains (*M. jejuensis* KCTC 32230, *C. flocculans* KCTC 62943, and *E. coli*, dh5α) and the microalgal strain *C. sorokiniana* KNUA114 were prepared. *C. sorokiniana* KNUA114 was isolated from Okcheon Stream in Ulleung, Korea (Okcheon Stream, Ulleung-eup, Ulleung-gun, Gyeongsangbuk-do; 37°28′ 24.0″ N, 130°53′ 06.8″ E; Yun et al., 2020). The bacterial and algal strains were maintained at 30°C on R2A agar plates composed of the following: proteose peptone, 0.5 g/L; casamino acid, 0.5 g/L; yeast extract, 0.5 g/L; dextrose, 0.5 g/L; soluble starch, 0.5 g/L; sodium pyruvate, 0.3 g/L; K_2_HPO_4_, 0.3 g/L; and MgSO_4_·7H_2_O, 0.05 g/L. For the experiments, each strain was cultured in R2A medium for 4 days at 30°C on an orbital shaker at 160 rpm in an incubation room. Cells were collected by centrifugation (4,000 rpm, 10 min) and washed three times with sterile distilled water. The initial cell density of the *C. sorokiniana* culture was set to an OD of 0.3 at 680 nm (OD_680_). Bacterial seeds were resuspended in fresh R2A medium (OD_600_ = 0.3). Each prepared cell sample (15 mL) was inoculated into 150 mL fresh R2A medium in a 250 mL flask. To compare the algal yields to bacterial flocs, six experimental groups (M, *M. jejuensis* only; C, *C. flocculans* only; E, *E. coli* only; MC, *M. jejuensis* and *C. sorokiniana*; CC, *C. flocculans* and *C. sorokiniana*; and EC, *E. coli* and *C. sorokiniana*) and one control group (N, *C. sorokiniana* only) were prepared. The experimental groups were cultured in heterotrophic conditions for 4 days at 30°C on an orbital shaker at 160 rpm in an incubation room.

### Flocs Size and Sedimentation Analysis

Floc size and its relationship to sedimentation rate were measured. The sizes of the floc colonies formed in the culture medium were measured using an optical microscope (model BX53F; Olympus). Samples were collected from 4-day-old cultures; 20 floc colonies were measured for each experimental condition.

To overcome the limitations of the existing sedimentation measurement using an Imhoff cone, the dry weight was used compared to separating the supernatant and the sediment (Sojka et al., 1992). For the sedimentation rate analysis, 4-day-old samples from each experimental condition were prepared, and 100 mL of each prepared sample were transferred to a glass container. The samples were shaken by hand until thoroughly mixed, and then 80 mL of supernatant was removed to obtain the lower 20 mL layer containing the sediment. The weight of cells contained in the supernatant and sediment was measured using the dry weight method (Yoo et al., 2010). In brief, a cell pellet was obtained using a pre-weighed glass-fiber filter and then dried in a drying oven at 105°C for 1 day before weighing (Yoo et al., 2010). This process was repeated for each experimental condition and measurement time.

### Biomass Collection and Lipid Content Analysis

Four-day-old cells were harvested by centrifugation at 4,000 rpm for 10 min. The collected cells were freeze-dried for 7 days, then ground using a mortar. The sulfo-phospho-vanillin reaction method was used to determine the total lipid content of each sample by mixing 10 mg of ground sample with 1 mL distilled water (Mishra et al., 2014). Different volumes of the mixture (10, 20, 30, 40, and 50 μL) were transferred to glass tubes and distilled water was added to achieve a total volume of 100 μL in each tube. Sulfuric acid (2 mL) was added to each of the glass tubes, which were then heated in a water bath at 100°C for 5 min. The tubes were allowed to cool to room temperature. Once cool, 5 mL phospho-vanillin reagent was added to each of the glass tubes, which were then shaken at 200 rpm in an incubator at 37°C for 15 min. The OD_530_ values of the mixtures were measured and total lipid content was calculated using canola oil as a reference standard (Mishra et al., 2014). To compare the fatty acid compositions between culture conditions, fatty acids were extracted from 30 mg of ground sample using a mixture of chloroform and methanol (1:1) (Yeo et al., 2011). The chloroform was removed from the fatty acid extract using an evaporator and methanol and potassium hydroxide were added to the mixture to facilitate transesterification of any lipids. Hexane was added to the extracted fatty acid mixture to isolate fatty acid methyl esters (FAMEs). The separated mixture was heated in a water bath at 70°C for 3 h. A gas chromatograph (model 6890N, Agilent) was used to analyze the FAME composition of the pre-treated samples (Furuhashi and Weckwerth, 2013).

### Statistical Analysis

We expressed the ratio of biomass present and the fatty acids contents, which we defined as 100%. We compared individual data points using Student's *t*-test, and a *P*-value of < 0.05 was considered statistically significant. All experiments were performed at least in triplicate, and the general microbiology test data were expressed as mean ± standard deviation (SD) (*n* = 3).

## Results and Discussion

### Whole-Genome Sequencing and Expected Genes for Flocculation

The characteristics of *M. jejuensis* are described in Tables 1, 2, and Supplementary Figure 1. The complete genome of *M. jejuensis* was comprised of a 3,754,826-bp chromosome, genome coverage was 195.39X, and G+C content was 67.5%. The genome contained 3,472 genes, including 3,380 protein-coding genes (CDSs), 30 pseudogenes, nine rRNA genes, and 53 tRNA genes. Total CDSs were matched to the EggNOG database (Powell et al., 2014). The *M. jejuensis* genome included eight aminopeptidase-coding genes, among which there was one ywaD gene (a leucyl aminopeptidase coding gene), which has been shown to control flocculation (Table 2; Geesey and Van Ommen Kloeke, 2004; Zhao et al., 2018). The genome also contained 17 polysaccharide biosynthesis protein-coding genes, including two UTP–glucose-1-phosphate uridylyltransferases (Table 2), which synthesize the EPS that form floc (Degeest and De Vuyst, 2000). Conversely, *C. flocculans* and *E. coli* each possessed only one UTP–glucose-1-phosphate uridylyltransferase-coding gene and no flocculation-controlling *ywaD* genes.

**Table 1:** General characteristics of *M. jejuensis, C. flocculans*, and *E. coli*, including flocculation-related genes.

**Property**	***M. jejuensis***	***C. flocculans***	***E. coli***
**Genome assembly**			
Assembly method	PacBio SMRT Link 7.0.1, SMRT Portal 2.3.0	RS HGAP Assembly version 3.0	M13 Janus shotgun strategy
Genome coverage	195.39X	364X	–
**Genome features**			
Genome size	3,754,826	3,333,437	4,641,652
G+C content	67.5	68.04	50.8
No. of contig	2	1	0
Total genes	3,472	3,197	4,583
Protein-coding genes	3,380	3,079	4,242
Pseudogenes	30	57	147
rRNA genes	9	9	22
tRNA genes	53	49	86
Aminopeptidase	8	5	7
Polysaccharide synthesis	17	7	3
EPS synthesis protein	2	1	1
Glycosyltransferase	9	8	5
References		Kim et al., 2019	Blattner et al., 1997

**Table 2:** List of predicted aminopeptidase and polysaccharide synthesis genes related to flocculation in *M. jejuensis, C. flocculans*, and *E. coli*.

**Strain**	**Gene group**	**Gene name**	**Product**	**Function**	**EggNOG no/. accession no**.
*M. jejuensis*	Aminopeptidase	*map*	Methionyl aminopeptidase	Aminopeptidase; hydrolase; metal-binding; protease	J:COG0024
		*map*	Methionyl aminopeptidase	Aminopeptidase; hydrolase; metal-binding; protease	J:COG0024
		*pepN*	Membrane alanyl aminopeptidase	Aminopeptidase; hydrolase; metal-binding; metalloprotease; protease; zinc	E:COG0308
		*dmpA|dap*	D-stereospecific aminopeptidase	Aminopeptidase; cell membrane; hydrolase; membrane; protease; transmembrane; transmembrane helix	EQ:COG3191
		*ywaD*	Aminopeptidase B	Carboxypeptidase; glycoprotein; hydrolase; protease; secreted; signal; virulence	E:COG2866
		*CARP|pepA*	Leucyl aminopeptidase	Aminopeptidase; cytoplasm; hydrolase; manganese; metal-binding; protease	E:COG0260
		*pip*	Prolyl aminopeptidase	Aminopeptidase; cytoplasm; hydrolase; protease	E:ENOG410XPKQ
		*pepP*	Xaa-Pro aminopeptidase	Aminopeptidase; cytoplasm; hydrolase; manganese; metal-binding; metalloprotease; protease	E:COG0006
	Polysaccharide synthesis	*lpxL|htrB*	Kdo(2)-lipid IV(A) lauroyltransferase	Acyltransferase; cell inner membrane; cell membrane; lipopolysaccharide biosynthesis; membrane; stress response; transferase; transmembrane; transmembrane helix	M:COG1560
		*lpxL|htrB*	Kdo(2)-lipid IV(A) lauroyltransferase	Acyltransferase; cell inner membrane; cell membrane; lipopolysaccharide biosynthesis; membrane; stress response; transferase; transmembrane; transmembrane helix	M:COG1560
		*UGP2|galU|galF*	UTP–glucose-1-phosphate uridylyltransferase	Capsule biogenesis/degradation; EPS synthesis; nucleotidyltransferase; Transferase	M:COG1210
		*UGP2|galU|galF*	UTP–glucose-1-phosphate uridylyltransferase	Capsule biogenesis/degradation; EPS synthesis; nucleotidyltransferase; Transferase	M:COG1210
		*kdsB*	3-Deoxy-manno-octulosonate cytidylyltransferase	Cytoplasm; lipopolysaccharide biosynthesis; nucleotidyltransferase; transferase	M:COG1212
		*OXCT*	3-Oxoacid CoA-transferase	Lipopolysaccharide biosynthesis; transferase	I:COG2057
		—	Lipopolysaccharide assembly protein	Cell inner membrane; Cell membrane; iron; membrane; metal-binding; Repeat; TPR repeat; transmembrane; transmembrane helix	G:COG2956
		*eptA|pmrC*	Lipid A phosphoethanolamine transferase	Antibiotic resistance; cell inner membrane; cell membrane; lipid a biosynthesis; lipid biosynthesis; lipid metabolism; lipopolysaccharide biosynthesis; membrane; transferase; transmembrane; transmembrane helix	S:COG2194
		*kdsA*	3-Deoxy-8-phosphooctulonate synthase	Cytoplasm; lipopolysaccharide biosynthesis; transferase	M:COG2877
		*waaE|kdtX*	Lipopolysaccharide core biosynthesis glycosyltransferase KdtX	Glycosyltransferase; lipopolysaccharide biosynthesis; transferase	M:COG0463
		*kdsC*	3-Deoxy-manno-octulosonate-8-phosphatase	Hydrolase; lipopolysaccharide biosynthesis; magnesium; metal-binding.	S:COG1778
		*rfbA|rffH*	Glucose-1-phosphate thymidylyltransferase	Lipopolysaccharide biosynthesis; magnesium; metal-binding; nucleotide-binding; nucleotidyltransferase; transferase	M:COG1209
		*rfbG*	CDP-glucose 4,6-dehydratase	Lipopolysaccharide biosynthesis; lyase; NAD	M:COG0451
		*per|rfbE*	GDP-perosamine synthase	Aminotransferase; lipopolysaccharide biosynthesis; pyridoxal phosphate; transferase	E:COG0399
		*rfbD|rmlD*	dTDP-4-dehydrorhamnose reductase	Lipopolysaccharide biosynthesis; magnesium; metal-binding; NADP; oxidoreductase	M:COG1091
		*rfbA|rffH*	Glucose-1-phosphate thymidylyltransferase	Lipopolysaccharide biosynthesis; magnesium; metal-binding; nucleotidyltransferase; transferase	M:COG1209
		*rfbC|rmlC*	dTDP-4-dehydrorhamnose 3,5-epimerase	Isomerase; lipopolysaccharide biosynthesis	M:COG1898
*C. flocculans*	Aminopeptidase	—	Putative aminopeptidase	Peptidase s58 dmpa	COG3191
		*pepA*	Cytosol aminopeptidase	Presumably involved in the processing and regular turnover of intracellular proteins; catalyzes the removal of unsubstituted N-terminal amino acids from various peptides (by similarity)	COG0260
		*pepP*	Xaa-Pro aminopeptidase	Peptidase M24	COG0006
		*map*	Methionine aminopeptidase	Removes the N-terminal methionine from nascent proteins (by similarity)	COG0024
		*pepN*	Aminopeptidase N	Aminopeptidase	COG0308
	Polysaccharide synthesis	*lapB_1*	Lipopolysaccharide assembly protein B	Tetratricopeptide repeat protein	COG2956
		*fepE*	Ferric enterobactin transport protein FepE	Lipopolysaccharide biosynthetic process	COG3765
		–	Oligosaccharide flippase family protein	Polysaccharide biosynthesis protein	COG2244
		*capD*	UDP-glucose 4-epimerase	Polysaccharide biosynthesis protein	COG1086
		*pglF*	UDP-N-acetyl-alpha-D-glucosamine C6 dehydratase	Polysaccharide biosynthesis protein	COG1086
		*lapB_2*	Lipopolysaccharide assembly protein B	Type IV pilus biogenesis stability protein PilW	COG3063
		*gtaB*	UTP–glucose-1-phosphate uridylyltransferase	UTP–glucose-1-phosphate uridylyltransferase	COG1210
*E. coli*	Aminopeptidase	*map*	Methionine aminopeptidase	—	NC_000913.3
		*pepN*	Aminopeptidase N	—	NC_000913.3
		*ypdE*	Broad-specificity exoaminopeptidase	—	NC_000913.3
		*ypdF*	Aminopeptidase	—	NC_000913.3
		*pepB*	Aminopeptidase B	—	NC_000913.3
		*pepP*	Proline aminopeptidase P II	—	NC_000913.3
		*pepA*	Aminopeptidase A/I	—	NC_000913.3
	Polysaccharide synthesis	*wbbK*	Putative lipopolysaccharide biosynthesis protein	—	NC_000913.3
		*waaZ*	Lipopolysaccharide core biosynthesis protein WaaZ	—	NC_000913.3
		*waaS*	Lipopolysaccharide core biosynthesis protein	—	NC_000913.3
		*galF*	UTP:glucose-1-phosphate uridylyltransferase, low activity	—	NC_000913.3

*Unclassified gene names and functions are replaced with an em-dash (—)*.

Based on the whole-genome sequencing results, *M. jejuensis* (aminopeptidase, eight genes; polysaccharide synthesis, 17 genes) possesses more flocculation-related genes than *C. flocculans* (aminopeptidase, five genes; polysaccharide synthesis, seven genes; Kim et al., 2019) and *E. coli* (aminopeptidase, seven genes; polysaccharide synthesis, three genes; Blattner et al., 1997). Furthermore, the gene expected to regulate flocculation, i.e., the *ywaD* gene (leucyl aminopeptidase-coding gene), was identified in *M. jejuensis* only. The UTP–glucose-1-phosphate uridylyltransferase gene involved in EPS synthesis was more abundant in *M. jejuensis* (two genes) than in *C. flocculans* (one gene) and *E. coli* (one gene; Table 1). Therefore, we expect rapid and efficient flocculation by *M. jejuensis* (Lee et al., 2013). Based on these results, we suggest that *M. jejuensis* has the potential to flocculate more efficiently than *C. flocculans* and *E. coli*.

### Flocculation and Sedimentation of Bacterial and Algal Flocs

**Figure 1:**
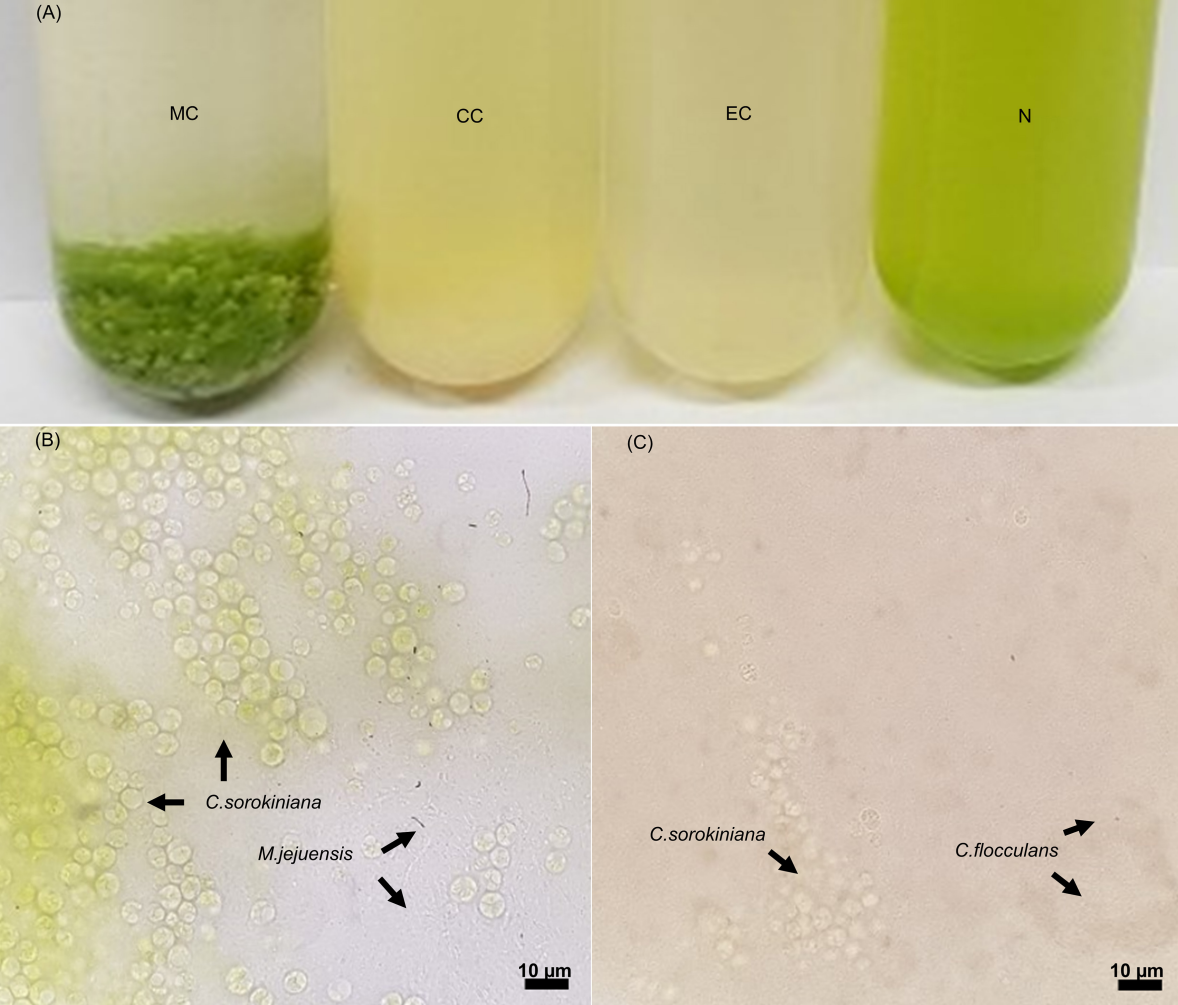
Images of cultures and light microscopy images of floc. In co-culturing conditions **(A)**, floc was formed in the MC and CC conditions, but no floc was observed in the EC and N conditions. The floc formed in the MC **(B)** and CC **(C)** conditions contained bacteria and algae (scale bar, 10 μm). The location of each species is marked with an arrow. MC, *M. jejuensis* and *C. sorokiniana*; CC, *C. flocculans* and *C. sorokiniana*; EC, *E. coli* and *C. sorokiniana*; N, *C. sorokiniana* only, control group. Marked characteristics are annotated in Supplementary Figure 1.

The co-culture of bacteria and algae resulted in the formation of both bacterial and algal flocs in some culture conditions (Figure 1). In the MC condition in which *M. jejuensis* and *C. sorokiniana* were cultured together, a green-colored floc of a visually recognizable size was observed; this floc was composed of tightly connected bacteria and algae. A floc composed of bacteria and algae also formed in the CC condition, where *C. flocculans* and *C. sorokiniana* were co-cultured. However, no flocs formed in the EC condition, where *E. coli* and *C. sorokiniana* were cultured together, or in the pure culture of *C. sorokiniana* (N condition). Based on previous studies, the cause of the formation of floc composed of microalgae and bacteria was expected to be from bacteria-derived materials, including EPS, and it was suggested that the difference in the material produced by each bacteria would be involved in the formation and characteristic of floc (Jimoh et al., 2019).

The supernatant in the MC condition became transparent immediately after shaking, although no particular difference between the turbidity of the supernatant and the lower layer was observed in the cultures of the other conditions (CC, EC, and N), demonstrating a greater biomass sedimentation ability in the MC condition. To understand the improved sedimentation ability observed in the MC condition, we measured the floc size as a trait related to sedimentation (Stone and Krishnappan, 2003; Bainbridge et al., 2012). The average floc size in the MC condition (4988.46 ± 2589.81 μm) was about 10 times larger than that in the CC condition (488.60 ± 226.22 μm). The size of floc obtained from MC and CC conditions exceeded that of pure microalgae cells, and this size was expected to have a large influence on sedimentation (Cheng et al., 2011; Baroni et al., 2019; Zhang et al., 2019).

**Figure 2:**
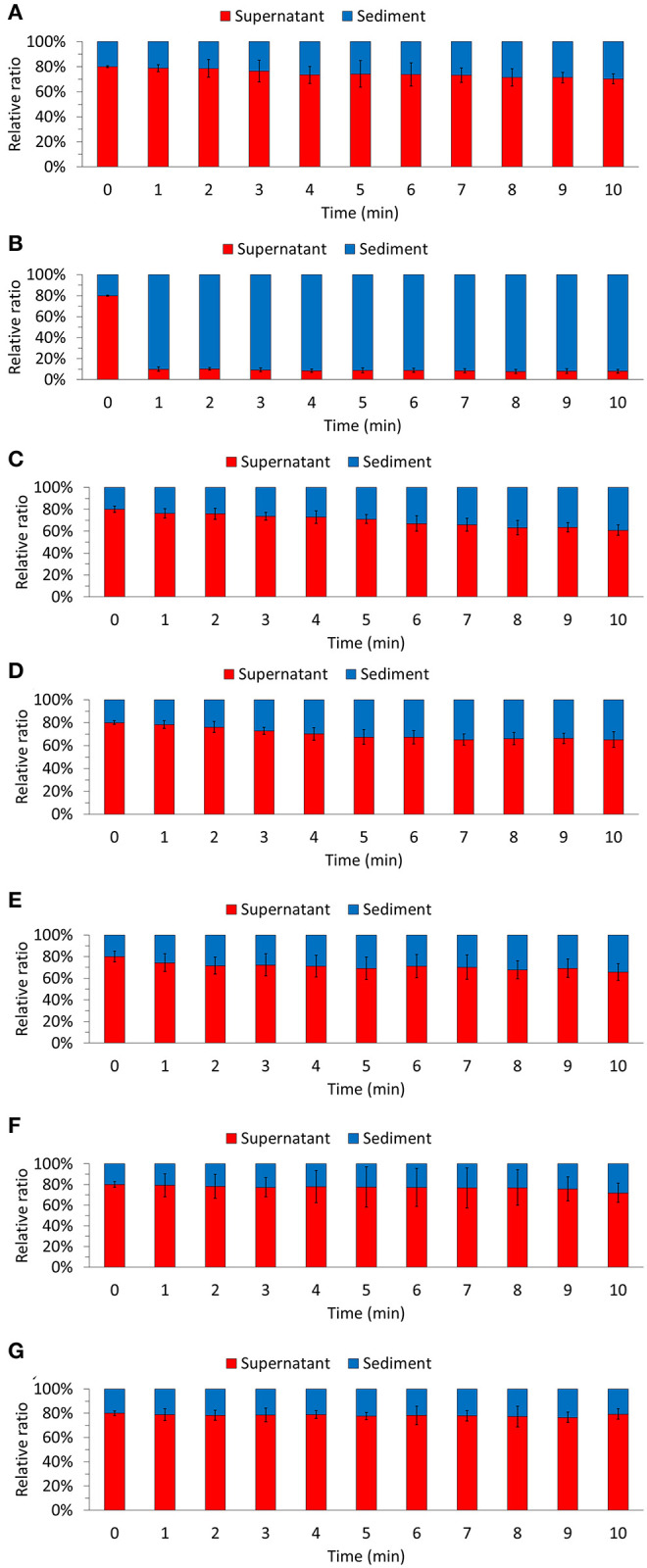
Biomass sedimentation ability was evaluated for each of the culture condition. N **(A)**, MC **(B)**, *M. jejuensis* **(C)**, CC **(D)**, *C. flocculans* **(E)**, EC **(F)**, and *E. coli* **(G)**. MC, *M. jejuensis* and *C. sorokiniana*; CC, *C. flocculans*, and *C. sorokiniana*; EC, *E. coli* and *C. sorokiniana*; N, *C. sorokiniana* only, control group. Marked characteristics are annotated in Supplementary Figure 1. We herein expressed the ratio of biomass present, which we defined as 100%.

Furthermore, we evaluated the biomass sedimentation ability of each culture (Figure 2). Considering the biomass of the whole culture, we compared the distribution of the sedimented biomass and the biomass contained in the supernatant. The highest sedimentation rates were observed in the MC condition (91.08 ± 2.32%). Although floc formed in the CC condition (32.55 ± 6.33%), there was no remarkable difference in the sedimentation rate compared to that in the EC condition (22.36 ± 19.52%) and the N condition (25.80 ± 10.53%). Our results demonstrate that the co-culture of bacteria and algae resulted in floc formation, and the highest biomass sedimentation rate was associated with large floc size. Therefore, it is expected that the sedimentation ability of the biomass produced by co-culturing the bacterial strain *M. jejuensis* KCTC 32230 with *C. sorokiniana* KNUA114 will be improved.

The floc formation by bacteria and algae improved sedimentation ability and reduced turbidity in a short period (Figure 2). Floc size is one of the main contributing factors affecting sedimentation ability (Stone and Krishnappan, 2003; Bainbridge et al., 2012). We also observed that the settleability improved in proportion to the floc size. In the MC condition, the sedimentation rate was 50 to 60% higher than in the CC condition. In addition, the floc-forming CC condition had a higher sedimentation rate (32.55 ± 6.33%) than the EC condition (22.36 ± 19.52%) and N condition (25.80 ± 10.53%), in which floc was not formed. Furthermore, when comparing the EC and N conditions, algae (*C. sorokiniana*), which has a larger cell size than bacteria (*E. coli*), exhibited a relatively high sedimentation ability. Based on the measured sedimentation ability and rate, we confirmed that floc formation affected biomass sedimentation. Therefore, our results suggest that biomass sedimentation efficiency can be improved by co-culturing bacteria and algae. Furthermore, our results support the potential application of *M. jejuensi*s KCTC 32230, which formed the largest floc, for biomass production.

### Pure Culture and Co-culture Growth Comparison

**Figure 3:**
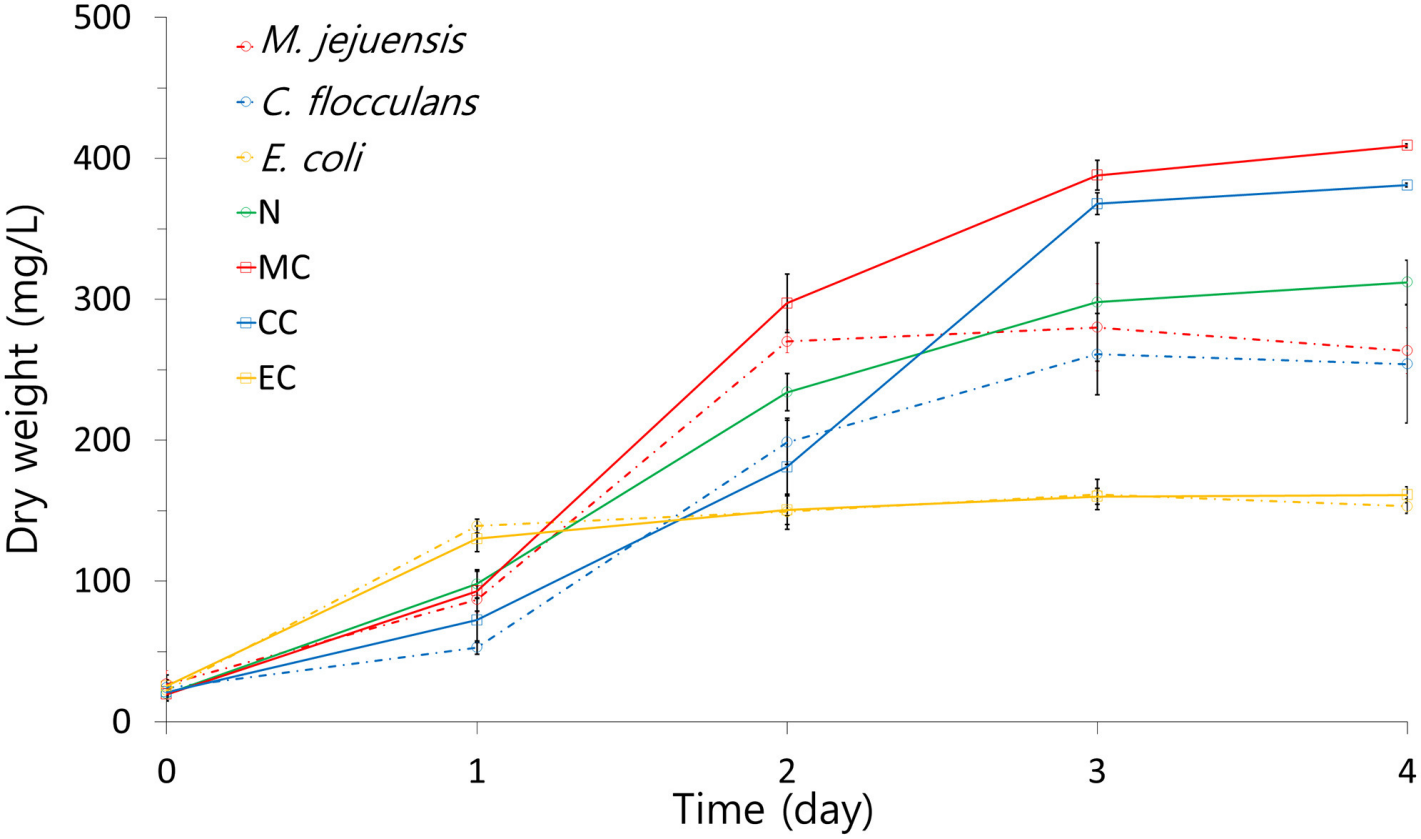
The growth pattern of each culture condition was visualized using dry weight. MC, *M. jejuensis* and *C. sorokiniana*; CC, *C. flocculans* and *C. sorokiniana*; EC, *E. coli* and *C. sorokiniana*; N, *C. sorokiniana* only, control group. Marked characteristics are annotated in Supplementary Figure 1.

The growth patterns of co-culture and pure culture are summarized in Figure 3. The highest dry weight was measured in the MC condition (409.00 ± 1.41 mg/L). The CC condition (381.00 ± 1.41 mg/L) also had a higher dry weight than the N condition (327.00 ± 20.09 mg/L), but the EC condition (163.87 ± 16.48 mg/L) did not. Additionally, the pure algae culture (N condition) had a higher dry weight than all pure bacteria cultures (*M. jejuensis*, 280.00 ± 30.99 mg/L; *C. flocculans*, 265.00 ± 21.79 mg/L; and *E. coli*, 161.3 ± 10.74 mg/L). The time taken to reach the stationary phase was the shortest in the EC condition (1 day) and pure *E. coli* culture (1 day). The stationary phase was reached in 2 to 3 days in the rest of the conditions and strains (*M. jejuensis*, 2 days; *C. flocculans*, 3 days; N condition, 3–4 days; MC condition, 3–4 days; and CC condition, 3–4 days). These results revealed that the co-culture of bacteria and algae had higher growth than pure bacterial and algal cultures.

Recently, studies have reported the application of heterotrophic cultivation methods for *Chlorella* (Fan et al., 2020). Studies have described microalgal species with high sedimentation and methods for improving microalgae harvesting efficiency through flocculation (Demir et al., 2020). Our research was conducted with wastewater and heterotrophic cultivation approaches in mind. Thus, we tried to induce microalgae floc using wastewater-derived or -related bacteria. *Chlorella* was selected as an experimental microalgal strain due to its smaller cell size than other microalgae, contributing to low sedimentation rates (Cheng and Liu, 2020; Demir et al., 2020; Potocar et al., 2020). Low sedimentation rate is the main limitation for biomass production from *Chlorella* (Cheng and Liu, 2020; Demir et al., 2020; Potocar et al., 2020); therefore, improving sedimentation can increase the usefulness of *Chlorella*. Various approaches to improving sedimentation have been attempted (Cheng and Liu, 2020; Demir et al., 2020; Potocar et al., 2020). Furthermore, industrial-scale facilities, such as wastewater treatment plants, require floc-forming and pollutant removal capabilities to remove pollutants. Sludge can be separated efficiently through floc-forming, and water can be purified through the ability of microalgae to remove nitrogen and adsorb heavy metals. Therefore, the floc-forming ability of bacteria and algae is important for the application of industrial-scale facilities (Chatsungnoen and Chisti, 2019; Sajana et al., 2019; Kim and Kwak, 2020; Zhou et al., 2020).

Comparing the growth trends of bacteria and algae co-cultures to pure cultures, better results were observed in all co-cultures, except *E. coli* (Figure 3). The fact that both MC and CC conditions reached a higher dry weight than the N condition suggests that co-culturing enables more efficient biomass production than algae alone cultured in the same conditions (Cho et al., 2015). However, based on the *E. coli* co-culture results, not all strains are suitable for co-culture. These growth-related results support improved growth by co-culture of *M. jejuensis* and *C. flocculans* with *C. sorokiniana*; *M. jejuensis* has the greatest potential to produce biomass efficiently.

### Co-culture Effects on Biomass Productivity and Lipid Quality

**Figure 4:**
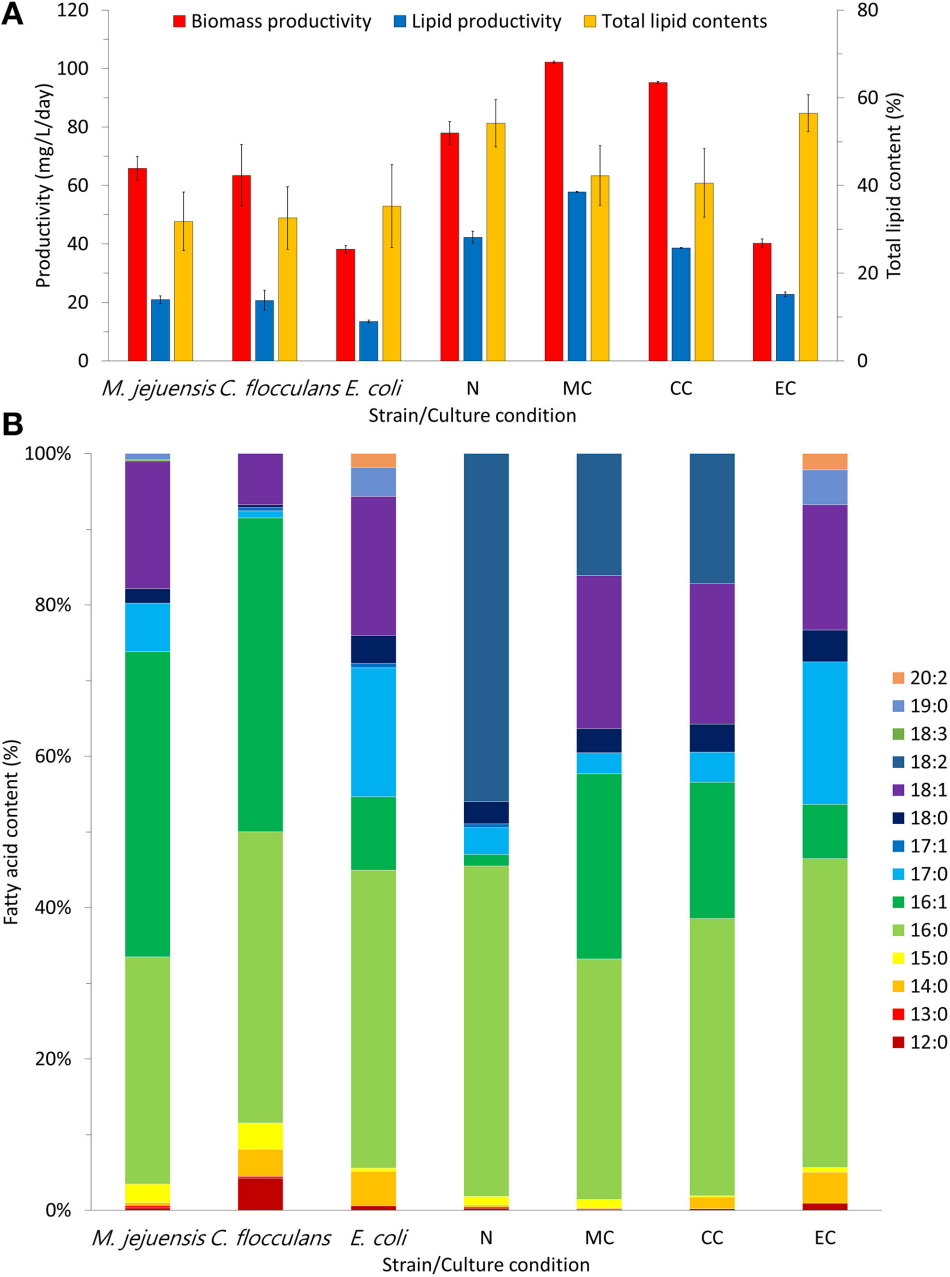
Productivity and fatty acid composition in each culture condition. The productivity of biomass and lipid content in each culture condition was measured **(A)**. Through fatty acid composition analysis, we confirmed the quality of lipids that were produced in each condition **(B)**. MC, *M. jejuensis* and *C. sorokiniana*; CC, *C. flocculans* and *C. sorokiniana*; EC, *E. coli* and *C. sorokiniana*; N, *C. sorokiniana* only, control group. We herein expressed the fatty acids contents, which we defined as 100%.

Biomass productivity was enhanced through the co-culture of bacteria and algae; the measurement results are summarized in Figure 4A. Among the tested conditions, MC had the highest biomass productivity [102.25 ± 0.35 mg/(L·day)]. The biomass productivity of the CC and N conditions was 95.25 ± 0.35 and 78.00 ± 3.89 mg/(L·day), respectively. Unlike the MC and CC co-cultures, which had enhanced biomass productivity compared to the N condition, the EC co-culture produced the lowest biomass of 40.25 ± 1.41 mg/(L·day). Furthermore, co-culturing improved not only biomass productivity, but also lipid productivity (Figure 4A). The MC condition showed the highest lipid productivity [57.80 ± 0.20 mg/(L·day)] in addition to the highest biomass productivity. Conversely, in the CC condition, which showed improved biomass productivity compared to that of the N condition, lipid productivity was inhibited [CC condition, 38.63 ± 0.14 mg/(L·day); N condition, 42.26 ± 2.11 mg/(L·day)]. The EC condition had the lowest lipid productivity at 22.75 ± 0.80 mg/(L·day). Therefore, the MC co-culture was the only condition in which both biomass and lipid productivity improved.

Additionally, we measured the changes in biomass lipid composition after improved productivity (Figure 4B). The biomass lipids obtained from co-culture (in MC and CC conditions) consisted mainly of C16:0 (MC condition, 31.80%; CC condition, 36.66%), C16:1 (MC condition, 24.45%; CC condition, 18.00%), C18:1 (MC condition, 20.23%; CC condition, 18.6%), and C18:2 (MC condition, 16.11%; CC condition, 17.14%). The biomass lipids obtained from the pure algal culture (N condition) consisted mainly of C16:0 (43.67%) and C18:2 (45.99%). Notably, the biomass lipids in the EC condition were mainly C16:0 (40.83%), C17:0 (18.80%), and C18:1 (16.57%); the composition was similar to that of pure *E. coli* (C16:0, 39.37%; C17:0, 17.05%; C18:1, 18.36%). Furthermore, when comparing the experimental groups (MC and CC conditions) to the respective pure bacterial biomass (*M. jejuensis* and *C. flocculans*) composed mainly of C16:0 (*M. jejuensis*, 30.01%; *C. flocculans*, 38.46%), C16:1 (*M. jejuensis*, 40.35%; *C. flocculans*, 41.51%), and C18:1 (*M. jejuensis*, 16.90%; *C. flocculans*, 6.74%), C16:1 increased and C18:1 and C18:2 decreased in the experimental group. Thus, the lipids in the co-cultured biomass showed the intermediate characteristics of the pure bacterial and algal lipid compositions.

In heterotrophic conditions, where the cultures were completely dependent on the organic carbon source in the media, a larger amount of biomass was produced in the co-culture than in the pure algal culture (Figure 4A). This phenomenon is contrary to previous reports of competition-induced growth degradation caused by limited organic carbon sources (Yadav and Archer, 1988). However, microorganisms are known to have preferred organic carbon sources, and different types of organic carbon are available depending on the environment (Brückner and Titgemeyer, 2002; Abreu et al., 2012). In addition, some studies suggest that the medium obtained by culturing certain microbial species can be recycled (Loftus and Johnson, 2017; Wang et al., 2018; Li et al., 2019). Therefore, it is expected that there has been no competition to impede growth in the use of organic carbon sources among species in conditions in which higher biomass could be produced (Loftus and Johnson, 2017; Wang et al., 2018; Li et al., 2019). Furthermore, improved lipid productivity may be related to lipid accumulation due to nutrient starvation (Chu et al., 2020; Poh et al., 2020). These results suggest that, although bacteria and algae were competing for a limited energy source, biomass production was more efficient in the co-culture than the pure algal culture (Berthold et al., 2019). However, the total lipid contents of the MC and CC conditions, which showed enhanced biomass productivity, were lower than that of the N condition. Nevertheless, the improved lipid productivity in the MC condition could be attributed to the increased biomass productivity, which improved enough to overcome the lower total lipid contents, unlike the CC condition (Figure 4A). Additionally, the co-culture biomass value was between the total lipid contents of the pure bacteria and the algae-derived biomass, which is presumed because the co-culture biomass was composed of bacterial and algae-derived biomass together (Berthold et al., 2019; Demir et al., 2020). Moreover, the improvement of lipid productivity and biomass suggests that high-quality biomass can be produced through the co-culture of bacteria and algae. Considering the biomass lipid composition results, we observed no extreme dominance between bacteria and algae in co-culture (Figure 4B). The identified biomass of the co-culture mainly consisted of C16:0, C16:1, C18:1, and C18:2, which was similar to the lipid composition of the biomass derived from pure bacteria (C16:0, C16:1, and C18:1) and algae (C16:0 and C18:2) together. The main components are expected to be derived from bacteria (C16:0, C16:1, and C18:1) and algae (C16:0 and C18:2). However, improvement in biomass and lipid productivity cannot be expected from all bacteria (Safonova and Reisser, 2005). Among the tested strains, the biomass and lipid productivity of *E. coli* in the EC condition was the lowest among the measured values. Moreover, the lipid composition of the EC condition was similar to that of pure *E. coli*. Based on this fact, it is likely that the EC condition was dominated by *E. coli*; we can assume that *E. coli* inhibited algal growth and biomass productivity (Safonova and Reisser, 2005). Therefore, to improve biomass and lipid productivity through the co-culture of bacteria and algae, it is necessary to select the appropriate species. Based on our experimental results, *M. jejuensis* KCTC 32230 is the most suitable bacteria for co-culture with *C. sorokiniana* KNUA114 among the strains tested here. Furthermore, our research demonstrates the potential for efficient biomass production through the co-culture of bacteria and algae.

## Conclusion

In this study, we demonstrated the value of bacterial- and algal-derived biomass for improving the harvesting efficiency using bacterial and algal floc. Among the bacterial strains tested, *M. jejuensis* KCTC 32230 formed the largest floc with *C. sorokiniana*, with the highest sedimentation ability. Furthermore, the *M. jejuensis* KCTC 32230 co-culture improved biomass and lipid productivity compared with the pure algal culture. However, co-culturing with *C. flocculans* KCTC 62943 or *E. coli* dh5α did not increase the productivity of biomass or lipids. Therefore, *M. jejuensis* KCTC 32230 is the most suitable bacterial strain for biomass production through the co-culturing of bacteria and algae. Our research provides insights into the efficient production and harvesting of biomass through the co-culture of bacteria and algae and highlights the need for suitable strain selection.

## Data Availability Statement

The raw data supporting the conclusions of this article will be made available by the authors, without undue reservation.

## Author Contributions

Y-SK, J-GK, D-HK, and H-SY designed the project and were involved in the writing of the manuscript. Y-SK and J-GK wrote the manuscript. D-HK and H-SY carried out most of the experiments and analyzed the data. All authors contributed to the article and approved the submitted version.

## Conflict of Interest

The authors declare that the research was conducted in the absence of any commercial or financial relationships that could be construed as a potential conflict of interest.
