# Supplementary material for: Effects of Co-culture on Improved Productivity and Bioresource for Microalgal Biomass Using the Floc-Forming Bacteria Melaminivora Jejuensis
Source: Front Bioeng Biotechnol. 2020 Dec 18;8:588210. doi: 10.3389/fbioe.2020.588210 (PMC7775480; doi:10.3389/fbioe.2020.588210)
Supplement: Supplementary file 1 [file Data_Sheet_1.pdf]

Supplementary Materials for

Effects of Co-Culture on Improved Productivity and Bioresource for Microalgal Biomass Using the Floc-Forming Bacteria *Melaminivora jejuensis*

Dong-Hyun Kim[1†], Hyun-Sik Yun[2†], Young-Saeng Kim[3*], Jong-Guk Kim[1,4*]

[1] School of Applied Biosciences, Kyungpook National University, Daegu, 37224, Republic of Korea

[2] Department of Biology, College of Natural Sciences, Kyungpook National University, Daegu 41566, Republic of Korea

[3] Research Institute of Ulleung-do & Dok-do, Kyungpook National University, Daegu 41566, Republic of Korea

[4] School of Life Sciences and Biotechnology, BK21 Plus KNU Creative BioResearch Group, Kyungpook National University, Daegu, 41566, Republic of Korea

[†] These authors contributed equally to this work

*** Correspondence:**
Tel.: +82-53-950-5348
Young-Saeng Kim: kyslhh1228@hanmail.net
Jong-Guk Kim: kimjg@knu.ac.kr

Supplementary Figure S1

(A)

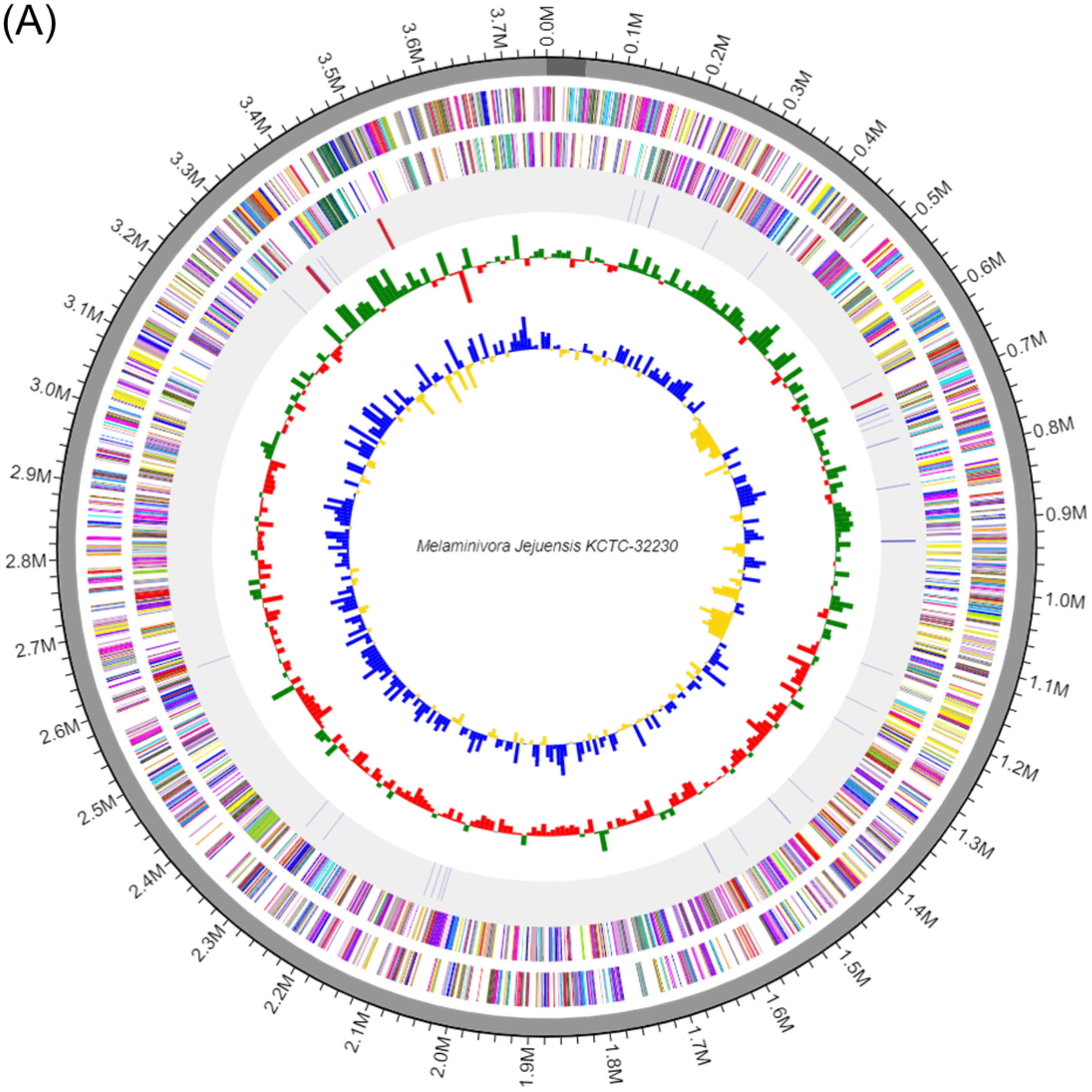

(B)

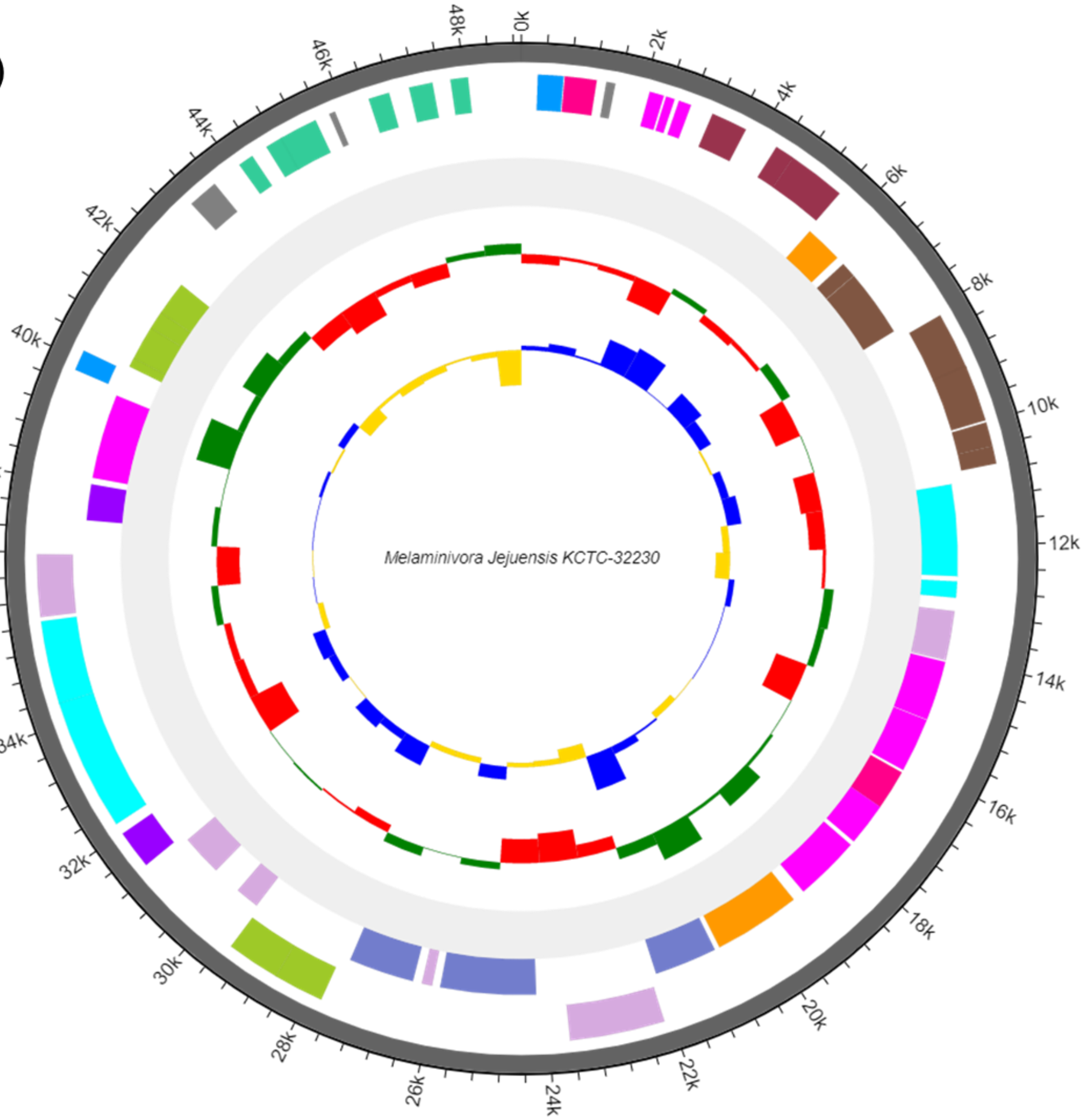
Melaminivora Jejuensis KCTC-32230
0k
2k
4k
6k
8k
10k
12k
14k
16k
18k
20k
22k
24k
26k
28k
30k
32k
34k
36k
38k
40k
42k
44k
46k
48k

(C)

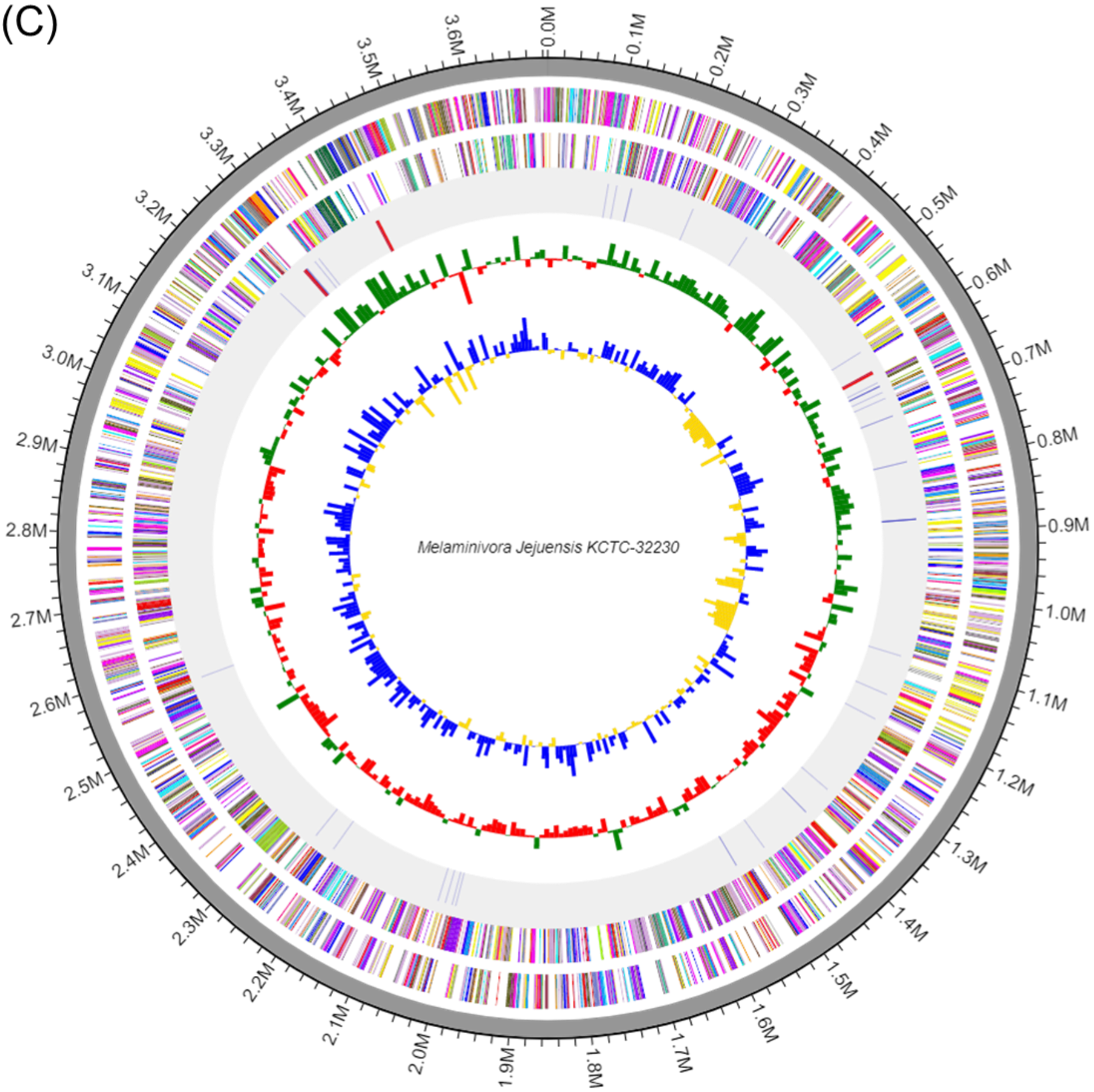

Supplementary Figure S1. Chromosome map of *M. jejuensis* KCTC 32230. Marked characteristics are shown from outside to the center; coding sequences on forward strand, coding sequences on reverse strand, Transfer RNAs (tRNAs), ribosomal RNAs (rRNAs), GC content, and GC skew. pseudo genome (A), contig 1 (B), contig 2 (C). Measurements are an annotated in Tables 1 and 2.
